# Topics of the Month

**Published:** 1894-09

**Authors:** 


### TOPICS OF THE MONTH.

There are more quacks in Buffalo than it has any reasonable use for, and every legitimate attempt to suppress them should not only receive the moral support of the profession, but the earnest and active coöperation of the constituted law authorities. A conviction or two for practising medicine illegally would serve to break up the nest of fiends that prey upon the unwary. Ignorant people visit the offices of these pretenders in large numbers and furnish almost their sole patronage. Uneducated people accept for truth the flaming advertisements in the newspapers and sown broadcast, especially when supported by pictures of the alleged successful cures. They, too, accept the statement of persons who claim to have been benefited by so-called treatment at the hands of these frauds, which is a sort of mouth-to-mouth method of advertising.

It should be the province of the medical profession to aid in the suppression of all illegitimate medical practice, and if the people cannot be enlightened on this subject, they should be prevented from being duped by the strong arm of the law.

Dr. J. F. Krug, chairman of the Board of Censors of the Medical Society of the County of Erie, has been doing some good work in the direction named, and he deserves not only the thanks of the profession, but to be aided in every way possible in the discharge of his official duties.

---

THE JOURNAL has heretofore with greater or less frequency given warning with reference to the dangers of the propagation of consumption, as well as spoken with reference to its prevention. Among other dangers that we have warned against is that of railway travel. While this menace is very great even in common railway coaches, it is increased many-fold in sleeping cars and especially in state rooms where invalids usually travel. The *Medical Record* recently published an article on this subject giving experiments made in the laboratory of the Imperial Board of Health of Germany. These investigations show that the seeds of consumption were found in abundance in the dust collected from the floors, walls and seats of railway carriages. Samples of dust were taken from forty-five compartments of twenty-one different cars and 117 animals were inoculated therewith. Part of these died very soon afterward of various contagious diseases before they had time to develop consumption. Of the rest, killed four to six weeks after inoculation, three had tubercles, and these were inoculated with sleeping car dust taken from the walls, cushions and ceilings.

We think it is high time that a rigid system of inspection should be inaugurated looking to the thorough cleansing and sterilizing of sleeping cars, and to the adoption of sanitary cars for day and night use. Especially do we urge the adoption of cars free from drapery, carpets and upholstery, and which shall be susceptible of being made aseptically clean after each tour of use.

---

Dr. Hermann M. Biggs, bacteriologist to New York Board of Health, was sent to Europe to investigate Koch's new discovery, which is alleged to be an infallible remedy for diphtheria. Dr. Biggs has made his report to the health department, in which he appears to justify all that has been said for the antitoxin cure. In this report he says :

> Out of more than 250 cases treated by the new method (the antitoxin), when the cases were inoculated on the first day, 100 per cent. recovered ; when treated on the second day, 97 per cent.; on the third day, 87 per cent.; on the fourth day, 76 per cent.; on the fifth day, 57 per cent. The conclusions seem to be justified that (1) any person after exposure can be rendered immune to the disease if the symptoms have not already developed. (2) If cases can be treated within the first thirty-six hours, or perhaps forty-eight hours, of the disease, the mortality may be reduced practically to zero. After this time the value of the treatment becomes progressively less.

The *British Medical Journal*, August 18, 1894, contains the details of three typical cases of diphtheria successfully treated with antitoxin.

---

While it is true that the good which men do lives after them, it is not always as promptly recognized as it should be. The entire profession of medicine in this country will ever feel grateful to the memory of Dr. John H. Rauch for what he did in advancing the standard of medical education while he was secretary of the Illinois State Board of Health, and for his labors in maintaining and improving the same after he retired from official life. We are pleased to note that Dr. R. H. Fitz, in his annual address before the Massachusetts Medical Society, paid graceful tribute to the memory of this distinguished publicist, as follows :

> There is not only a prolongation of the period of study as the effect of these laws, but there is also an increased demand for a preliminary education, the establishment of new professorships, and more exacting examinations for the degree. Of all agents distinctly bringing about this change, the Illinois State Board of Health, and especially its secretary, the late Dr. John H. Rauch, deserve the highest consideration.

---

Messrs. W. H. Schieffelin & Company, of New York, have published a handsome brochure, entitled One Hundred Years of Business Life. It is a simple and well-written history of the house of Schieffelin from 1794 to the present, and very appropriately adopts the motto, "*Respice, Adspice, Prospice.*" One hundred years of continuously successful business life is so unusual in the rapidly changing conditions that obtain in this country as, in our opinion, to justify this prosperous house in so appropriately celebrating its centennial. It has survived the disruptions of business incident to three great wars, and has equally successfully withstood the shock of as many financial panics, emerging each time stronger for the trials it had passed, and, reconstructing itself with new blood and enlarging its avenues of trade, stands today as one of the most prosperous houses of its kind, noted alike for its business integrity and its sound financial standing.

In this well-illustrated history is given biographical sketches of the heads of the house from time to time, and it closes with a fitting tribute to its subordinates. It is not uncommon for persons to serve the firm for twenty-five years, and it has in its employ now ten persons whose periods of service range from thirty to forty years, while one has remained the extraordinary period of forty-five years. Such a record speaks more eloquently than words for the sterling integrity of the house, which deserves the congratulations of its fellow-citizens on this centennial anniversary.

---

THE Buffalo State Hospital has suffered a severe blow in the death of Dr. J. B. Andrews, its able and accomplished superintendent. Dr. Andrews was appointed when the institution was organized, in 1880, and had served continuously until his death. Appropriate action on this sad event has been taken by the Medical Society of the County of Erie, full report of which is published in another column. Our purpose here is only to refer to the relations Dr. Andrews sustained to the State Hospital and to the effect produced on the institution by his death.

While no man is so necessary to the world but that his place, when vacated, can be filled more or less completely, yet it must be confessed that the managers of the Buffalo State Hospital are confronted with a difficult problem in the appointment of a successor to Dr. Andrews. He was remarkably well equipped for the high office he held, both in physical strength and in mental equipoise. A man of great amiability of character, profundity of learning, admirable executive powers and courteous demeanor, he was unusually well armed with all those qualities that go to make up a great alienist and a successful superintendent of a large hospital for the insane. We repeat that to select a successor to such a man is no easy task. However, under our present laws, the matter is much simplified, and there is every reason to believe that Dr. A. W. Hurd, who has been so many years Dr. Andrews' first assistant, and who has necessarily a large experience, will, if chosen, make an acceptable and satisfactory superintendent.

---

THE twenty-third annual report of the managers of the Buffalo State Hospital, the same being for the year 1893, is on our table. It is an interesting showing of the work of the year and should be examined by every one interested in the care of the insane. The wards are overcrowded, in spite of the recent extension, and new buildings are demanded. These are in process of construction and, probably, by another year will be ready for occupancy. These, when completed, will make the Buffalo hospital the largest and most complete institution of its kind in the country. Such a hospital deserves well at the hands of the State, as well as the moral support of the community.

---

THE *Maryland Medical Journal* makes a very sensible comment relating to the unkind words that often pass between medical journals, as follows:

> Medical journals are usually published as mirrors of thought and medical opinion of the country or state whence they emanate, and they should by all means be free from bickering and back-biting, and yet hardly a week passes but some are finding fault and looking for each other's weak points. This is not only unnecessary, but it is extremely distasteful to most readers, who are not seeking for gossip or private grievances in a scientific journal, but read that they may be benefited and instructed. It is only the poorer papers of a certain class that get their support by printing slanders against their neighbors, and all such personalities should be omitted from the columns of respectable medical journals.

We think it would be well for all of us to read this over occasionally, hence have reproduced it for the purpose of accentuating this able editor's excellent thought.

---

THE legislature of New York (*Maryland Med. Jour.*) has passed a bill establishing a pension fund for employes of the health department. This act provides for physicians, nurses, clerks, and other employes who have served a term of twenty years, and for the families of employes dying while in discharge of duty. The pensions will be paid from a fund created by the fines and penalties collected for violations of the city health laws and by fees for examinations of the records.

